# Successful Use of Extracorporeal Life Support after Double Traumatic Tracheobronchial Injury in a Patient with Severe Acute Asthma

**DOI:** 10.1155/2011/936240

**Published:** 2011-11-02

**Authors:** Xavier Valette, Aurélie Desjouis, Massimo Massetti, Jean-Luc Hanouz, Philippe Icard

**Affiliations:** ^1^Department of Anesthesia and Intensive Care, Centre Hospitalier Régional et Université de Caen Basse Normandie, Avenue Cote de Nacre, 14033 CAEN Cedex, France; ^2^Department of Thoracic and Cardiovascular Surgery, Centre Hospitalier Régional et Université de Caen Basse Normandie, Avenue Cote de Nacre, 14033 CAEN Cedex, France

## Abstract

We report the case of an asthmatic patient with blunt trachea and left main bronchus injuries who developed acute severe asthma after surgical repair. Despite medical treatment and ventilatory support, asthma persisted with high airway pressures and severe respiratory acidosis. We proposed venovenous extracorporeal life support for CO_2_ removal which allowed arterial blood gas normalization and airway pressures decrease. Extracorporeal life support was removed on day five after medical treatment of acute severe asthma. So we report the successful use of extracorporeal life support for operated double blunt tracheobronchial injury with acute severe asthma.

## 1. Introduction

Double tracheobronchial injuries are rare and may require prompt surgical repair [1, 2]. Immediate extubation is preferable to avoid the harmful effects of ventilation pressure on sutures. In such situations, severe acute asthma requiring mechanical ventilation presents a high risk of suture leakage because of sustained airway pressure. Here, we report a case of successful management of such a situation by extracorporeal life support (ECLS).

## 2. Case Report

A 17-year-old patient, who was a smoker with asthma, suffered a motorcycle accident. This patient had mild intermittent asthma with only occasional short-acting beta-agonist inhalations. At the scene, he rapidly developed extended subcutaneous emphysema (to the knees) with shortness of breath and a decrease in peripheral oxygen saturation, requiring immediate orotracheal intubation and mechanical ventilation. A computed tomography scan showed a bilateral pneumothorax with pneumomediastinum and pneumoperitoneum, a tracheal tear above the carina, a left main bronchus laceration below the carina, a left upper lobar pulmonary contusion, and a left hemothorax. 

In the emergency operating room, surgical repair of the trachea and left main bronchus was performed during a right posterolateral thoracotomy through the fifth intercostal space. The orotracheal intubation was not selective. The anterior tracheal tear, 3 cm above the carina, was repaired using 3 interrupted 4-0 Prolene suture covered over by mediastinal fat. On the posterior left main bronchus, 1.5 cm below the carina, 2 tears were repaired by 4.0 Prolene suture on the mucosae and the membranes. After surgery, the tracheobronchial tree was examined with endoscopy. One left and 2 right chest tubes were left in place postoperatively.

Immediately following surgery, we could not extubate the patient because he presented a bronchospasm that required high ventilation mechanical support in the intensive care unit. Despite the medical treatment used for acute asthma (nebulized terbutaline and ipratropium bromide, sedation by propofol and ketamine, systemic corticotherapy, intravenous magnesium sulfate and epinephrine infusion), protective mechanical ventilation resulted in a plateau airway pressure (Pplat) of 30 cm H_2_O, a peak airway pressure (Ppeak) of 60 cm H_2_O, and an intrinsic positive end expiratory pressure of 13 cm H_2_O. In contrast, the PaCO_2_ increased to 64.1 mmHg and arterial pH decreased to 7.16. Consequently, extracorporeal CO_2_ removal was proposed using venovenous extracorporeal life support (ECLS) device. It was performed at bedside using the Seldinger technique. A 19-Fr drainage cannula was inserted into the left femoral vein, and a 15-Fr return cannula was inserted into the right jugular vein. The activated clotting time was maintained between 175 and 200 seconds. Following initiation of ECLS, the arterial blood gas normalized (pH = 7.39 and PaCO_2_ = 34.5 mmHg). This allowed us to set the mechanical ventilation to decrease airway pressures (Pplat 22 cm H_2_O, Ppeak 45 cm H_2_O). Medical treatment for bronchospasm was continued with nebulized terbutaline and ipratropium bromide, whereas corticotherapy and epinephrine were stopped on day 3. Bronchoconstriction disappeared progressively, which made it possible to resume conventional ventilation with lower airway pressure on day 4. During this period, arterial blood gases were normalized through ECLS. 

The patient experienced 2 complications: he developed pneumonia on day 4 and a left hemothorax on day 5. At that time, ECLS was unnecessary because conventional ventilation could be done. ECLS was removed at bedside, then the hemothorax was evacuated during a left thoracotomy performed in the operating room because of the incapacity of drainage by chest tube. The patient was weaned from the ventilator on day 9 and was discharged to go home on day 21. The patient is now in good health, with no pulmonary disorder, and with stabilized asthma.

## 3. Discussion

**Figure 1:**
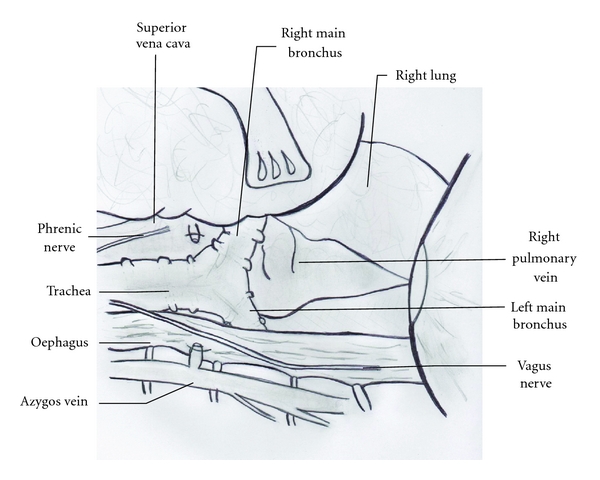
View of technical approach of the carina and the left main bronchus. On this view: right main bronchus, left main bronchus, trachea, right pulmonary vein, esophagus, azygos crosscut. A good way for posterior lesions of the trachea, left and right main bronchus.

To our knowledge this is the first case report of multiple tracheobronchial injuries associated with severe acute asthma, as tracheobronchial injuries are rare. A retrospective analysis from a level 1 trauma center reported that 0.13% of trauma patients had tracheobronchial injuries [3]. These injuries require rapid diagnosis, adequate airway management, and prompt surgical repair [1, 2]. Mortality rates of 15% to 18% have been reported [1, 2]. Delays in diagnosis and associated injuries have been suggested to be associated with mortality. Nonoperative treatment can be chosen when the patient is stable, with small and asymptomatic tracheobronchial injuries (less than one-third of the tracheal circumference) [2, 3]. Most injuries have been reported to be located 2.5 cm from the carina, and the right main bronchus has been shown to be more frequently damaged than the left (Figure 1) [1, 2]. 

Tracheal tears can also result from intubation. Iatrogenic tracheobronchial rupture (TBR) has different mechanisms leading to different morphologic appearance with classically longitudinal laceration of the posterior tracheal wall. Iatrogenic TBR may be managed operatively or nonoperatively [4]. In the present case, initial extended subcutaneous emphysema before orotracheal intubation and morphologic appearance of the lesions with multiple tracheobronchial injuries with damage to the left main bronchus, that are rare in iatrogenic TBR, are in favour of a traumatic origin.

A right posterolateral thoracotomy was undertaken because it is the usual surgical procedure for treating injuries or tumors of the proximal left main bronchus. The best surgical approach for mediastinal tracheal and bronchial disruption is right postero-lateral thoracotomy [2, 5]. Using this method, the surgeon can access the carina and left main bronchus at its origin. Regarding the anesthesia technique, intubation with a double-lumen tube is the most common and comfortable method for ventilating the contra lateral lung without air leakage during bronchial reconstruction in adult patients [6].

Severe acute asthma is life threatening and requires immediate adequate medical therapy. Mechanical ventilation should be avoided but is still required in about 30% of cases [7, 8]. The mortality rate has been reported to be 8% [7, 8]. In the present case, despite medical treatment for asthma, mechanical ventilatory support was required but resulted in strong airway pressure, which may be involved in tracheobronchial suture leakage or rupture. Furthermore, mechanical ventilation was ineffective in removing CO_2_ leading to severe respiratory acidosis.

In intensive care, ECLS was successful in rapidly removing CO_2_ and enabling us to set the protective mechanical ventilation thus decreasing airway pressure. ECLS is widely used to support patients with acute respiratory distress syndrome or with various types of acute reversible cardiac failure [9, 10]. Its use following tracheobronchial injury associated with asthma has never been described. In contrast, ECLS has been used in severely injured patients with refractory hypoxemia [11–13]. Similarly, ECLS has been used for patients with tracheobronchial injuries [14] and for severe acute asthma [8, 15].

The present paper strongly suggests that ECLS may help to successfully manage severe acute asthma occurring immediately after tracheobronchial injury repair. In such a situation, ECLS may help to limit airway pressures resulting from mechanical ventilation, which increases the risk of suture damages.
